# Influence of Temperature and Flow Rate on Erosion–Corrosion of Low-Alloy Steel in Simulated Steam Generator Conditions

**DOI:** 10.3390/ma18050944

**Published:** 2025-02-21

**Authors:** Martin Bojinov, Iva Betova, Nikoleta Ivanova, Vasil Karastoyanov

**Affiliations:** 1 Department of Physical Chemistry, University of Chemical Technology and Metallurgy, 8 Kliment Ohridski Blvd., 1756 Sofia, Bulgaria; n.ivanova@uctm.edu (N.I.); vasko_kar@uctm.edu (V.K.); 2 Institute of Electrochemistry and Energy Systems, Bulgarian Academy of Sciences, 1113 Sofia, Bulgaria; i.betova@iees.bas.bg

**Keywords:** flow-assisted corrosion, low-alloyed steel, steam generator coolant, electrochemical impedance spectroscopy, kinetic model

## Abstract

The erosion–corrosion mechanism of low-alloy steel in high-ammonia steam generator’s chemistry is studied by in situ impedance spectroscopy coupled with an in-depth analysis of formed oxides using glow discharge optical emission spectroscopy. A novel electrode setup that ensures turbulent conditions in the vicinity of the steel sample is used. The effect of temperature (130–230 °C) and flow rate (2–10 dm^3^ h^−1^) is investigated. The energy of adsorption of ammonia depends on temperature and is estimated using molecular dynamic simulations. The kinetic and transport parameters of the corrosion process are estimated via the regression of the experimental impedance spectra to the transfer function of the Mixed-Conduction Model for oxide films. Conclusions are drawn about the effect of Cr in the alloy, and the temperature and flow rate on the corrosion mechanism.

## 1. Introduction

Flow-accelerated corrosion (FAC) is an environmentally assisted degradation mode of carbon and low-alloy steel in de-aerated, alkaline, high-temperature water at large flow rates [1,2,3,4,5]. In FAC-favored environments, the deposit layer of magnetite (Fe_3_O_4_) is dissolved in bulk water by electrochemical reactions coupled with mass transport in turbulent conditions [6]. The continuous dissolution of the oxide layer leads to wall-thinning and, eventually, to pipe rupture. As a countermeasure, the replacement of carbon steel with low-alloy steel containing Cr and Mo has been suggested, since trace amounts of alloy elements can lead to a large decrease in FAC rates [7,8]. Historically, there has been substantial research and quantitative analysis of the influence of corrosion resistive alloy elements such as Cr, Cu, and Mo on both the general and localized corrosion resistance of alloys [9]. In the mid-nineties, a transient model to determine the impact of Cr on FAC was proposed [10]. The model indicated that low-alloy steel with Cr content > 0.04% becomes more protective with time of exposure.

However, there is still a lack of understanding of the quantitative evaluation of the FAC rates with regard to the oxidation and corrosion behavior of low-alloy steel under steam generator water chemistry. For carbon steel in single phase flow, it is known that levels of Cr as low as 0.1% can lead to a substantial decrease in the FAC rate [11]. The addition of Cr has been shown to significantly enrich the Fe_3_O_4_ layer and, eventually, promote a reduction in the dissolution rate in high-temperature alkaline water [12,13]. The primary mechanisms of FAC involve the simultaneous dissolution of the oxide film at the oxide/water interface and oxide formation reactions at the oxide/parent material interface, leading to the reduced thickness of the parent piping material [1,2,3]. The chemical composition of the oxide film is determined by the combined effects of the material and water chemistry factors, and it is also directly related to the oxide film’s solubility [14]. However, during continuous dissolution and oxidation in flow environments, the oxide layer can undergo changes in physical and electrochemical characteristics. Macroscopically, they are changes in shape and porosity, and microscopically, they are changes in atomic arrangement, crystalline structure, ion and electron transport rates. However, although recent studies have focused on assessing thickness variations through non-destructive analysis [15,16,17,18] or computational fluid dynamics analysis [19,20], most empirical treatments have been limited to assessing FAC rates using the weight loss method. More specifically, oxide films were only subject to simple analysis such as surface morphology comparisons, and in situ electrochemical methods have been scarcely employed [21].

The present paper is devoted to the characterization of FAC of low-alloy steel containing mainly Cr in the steam generator coolant (NH_3_ solution), covering a wide range of temperatures (130–230 °C) and flow rates (2–10 dm^3^ h^−1^), and using in situ electrochemical impedance spectroscopy (EIS) to assess the electric and electrochemical properties of the formed oxides. EIS is coupled with an ex situ characterization of the thickness and in-depth composition of corrosion films using Glow-Discharge Optical Emission Spectroscopy (GDOES). The quantitative interpretation of the EIS data using the Mixed-Conduction Model for oxide films (MCM) allowed for the estimation of the rates of interfacial charge transfer, ion, and electron transport through the growing corrosion film as depending on temperature and flow rate. A correlation between the FAC rate and charge transfer at the film/coolant interface is proposed, which is in good agreement with the relationship of thickness and Cr enrichment in the oxide. In addition, the adsorption energy of NH_3_ estimated via molecular dynamics simulations is discussed in terms of the role of ammonia in the corrosion mechanism of low-alloyed steel in the studied conditions.

## 2. Materials and Methods

**Figure 1:**
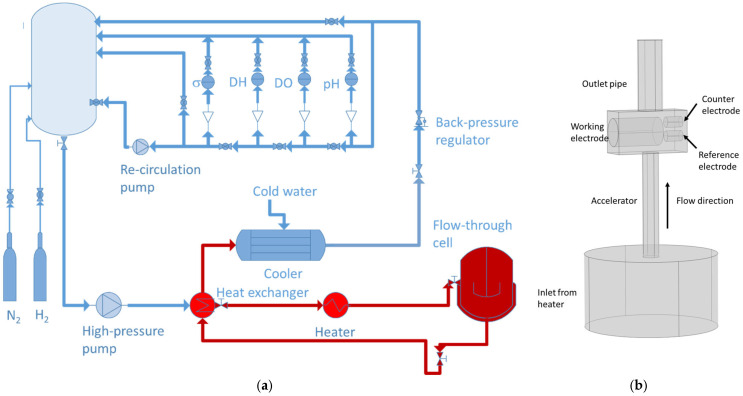
(**a**) Re-circulation loop with a flow-through cell (blue—low-temperature part, red—high-temperature part); (**b**) flow-through cell with accelerating insert to study FAC.

The experimental setup employed in the present investigation is shown in Figure 1a.

The low-temperature part of the loop consists of a 20 dm^3^ electrolyte reservoir equipped with valves for gas purging, an array of sensors to monitor the water chemistry via specific conductivity, pH, dissolved hydrogen (DH), and oxygen (DO) gases, a high-pressure and a recirculation pump, a back-pressure regulator, and a cooler. An array of heaters is installed in the hot part to reach a maximum temperature of 280 °C, together with a heat exchanger and a flow-through cell. A blow-up of that cell is shown in Figure 1b. It contains a flow accelerating pipe, a compartment for the working electrode—a low-alloy steel rod (composition, wt.%: 0.2% C, 0.6% Cr, 0.1% Ni, 0.1% Mn, 0.24% Si, balance Fe), counter and reference electrodes (Pt and Pd, respectively). The Pd electrode is polarized with small negative currents (10–30 μA) to approximate the reversible hydrogen electrode at a given condition. All the potentials are referred to the standard hydrogen electrode (SHE). The hydrodynamic conditions in the flow-through cell (linear velocity and Reynolds number) were calculated with the k-ε turbulent flow model via 3-D simulations with a finite-element method (Comsol Multiphysics 6.1, Burlington, MA, USA).

A typical experimental run comprised a 16 h purging of the electrolyte (a high-pH steam generator coolant, 0.08 mmol dm^−3^ NH_3_, room temperature pH of 9.9 ± 0.2, pressure 90 bars) with 99.999% N_2_ to reach a DO concentration less than 5 μg kg^−1^. Subsequently, the system was heated up to the respective temperature (130–230 °C). A flow rate of 2 dm^3^ h^−1^ was maintained for 24 h, followed by an increase to 10 dm^3^ h^−1^ for the next 24 h and finally a return to 2 dm^3^ h^−1^ in the last 24 h of the experiment.

Electrochemical impedance spectra were continuously measured by 10030 potentiostat with a frequency analyzer (Ivium, Eindhoven, The Netherlands) in a frequency range of 1.1 kHz to 0.1 mHz in a galvanostatic zero dc current mode using an ac current amplitude of 10 μA (rms) as the upper limit at which impedance was independent on amplitude (i.e., linearity was respected). The current signal resulted in a typical voltage perturbation of 60–70 mV (rms). Causality of the impedance was verified by application of a Kramers-Kronig compatibility test to selected data sets using the measurement model and associated software [22]. Non-linear regression of experimental impedance data to the transfer function of the Mixed-Conduction Model (MCM) is implemented as a complex fitting using Origin Pro 9.8 software (Originlab, Northampton, MA, USA).

Thickness and in-depth elemental composition of oxides on steel are estimated by GDOES (primary voltage 950 V, current 9 mA, and pressure 3 hPa) using a GD750HR apparatus (Spectruma Analytik, Hof, Germany). The reference materials used to calibrate the analysis were chosen to cover the elements in the relevant concentration ranges pertinent to low-alloy steels.

The adsorption of ammonia on Fe_3_O_4_ [111] in an aqueous phase was studied using molecular dynamics simulations via the Clay force field in a similar temperature range (25–200 °C) at a pressure of 90 bars. Periodic boundary conditions (PBCs) with changed dimensions along the z coordinate of 10 nm were imposed on the already built layer of Fe_3_O_4_. The number of water molecules in the aqueous phase was 3437 with 12 ammonia molecules, resulting in NH_3_ concentration of 1.8 × 10^−4^ mol dm^−3^. In the next step, the model system was saturated with water molecules until the correct density was reached for the given conditions. The root mean square deviation of the coordinates of the two types of iron ions and the dissolved molecules in the aqueous phase were also monitored. The trajectory length was 200 ns, with some results presented for the last 50 ns. For each temperature, four independent runs were made with different configurations for the ammonia and water molecules and with different velocities for the magnetite atoms, but the same initial coordinates in the model system. Molecular dynamics (MD) calculations were performed with the Large-scale Atomic/Molecular Massively Parallel Simulator (LAMMPS) August 2023 software package. Energy calculations were performed for the last 5 ns of the productive part of trajectories.

## 3. Results

### 3.1. Hydrodynamics and Water Chemistry

**Figure 2:**
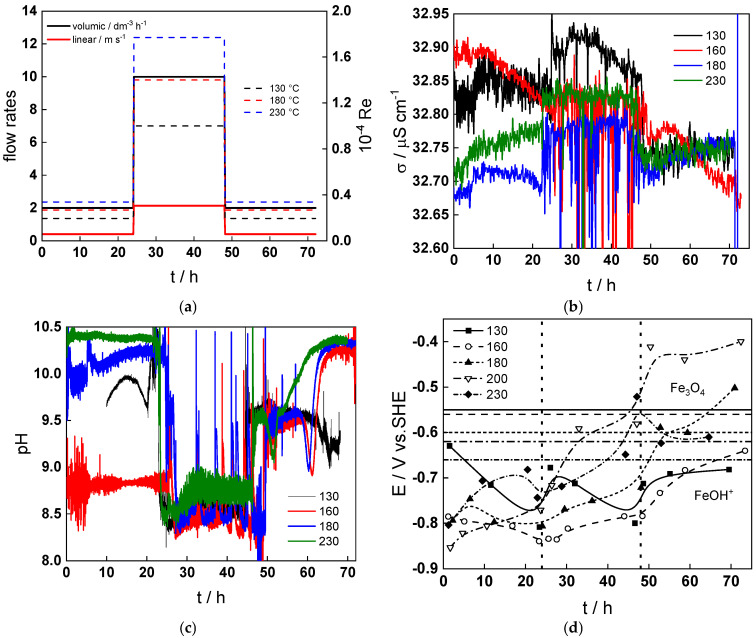
(**a**) Profiles of applied volume flow rate, calculated linear fluid rate, and Reynolds number with time during a typical experimental run, (**b**) specific conductivity vs. time during runs at different temperatures, (**c**) pH vs. time during runs and different temperatures, (**d**) corrosion potential as a function of time at all studied temperatures. The limit of thermodynamic stability of magnetite and FeOH^+^ at different temperatures is shown with horizontal lines, whereas the vertical lines indicate the transitions to turbulent regime and back.

Figure 2 illustrates the evolution of the water chemistry with temperature and flow rate. When the conditions are changed from a transition to turbulent regime (at 24 h), the conductivity of electrolyte (Figure 2b) increases most probably due to the formation of divalent iron ions via enhanced dissolution of the oxide and corrosion release. This suggestion is corroborated by the decrease in pH during that transition (Figure 2c). The transient dissolution process is more pronounced at temperatures lower than 180 °C, whereas at 230 °C it is to a certain extent reversible (i.e., after the flow rate is decreased, the values of conductivity and pH return to their original values).

The corrosion potential (Figure 2d) slightly increases when the regime is changed to turbulent at low temperatures (below 200 °C), which can be correlated to the alterations in conductivity and pH and the transient increase in oxide dissolution and corrosion release rates since in this temperature interval, FeOH^+^ is predicted to be the thermodynamically stable corrosion product. At higher temperatures, the increase in potential is more pronounced, especially after the regime is returned from turbulent to transitional (at 48 h). This means that passive layer formation is favored in agreement with the fact that Fe_3_O_5_ is the stable corrosion product in that temperature interval.

### 3.2. Electrochemical Impedance Spectroscopy

**Figure 3:**
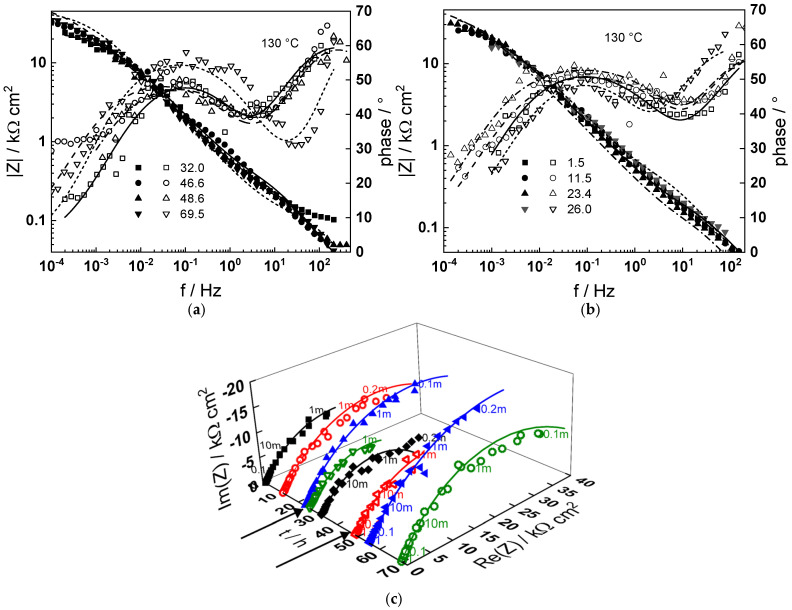
(**a**,**b**) Magnitude (left axis, full symbols) and phase angle (right axis, open symbols) of impedance as depending on frequency for different exposure times at 130 °C, (**c**) dependence of imaginary part of impedance on real part and time of exposure (parameter is frequency in Hz). Transitions to turbulent regime and back indicated with arrows. Points—experimental values, lines—best-fit calculation according to the MCM.

**Figure 4:**
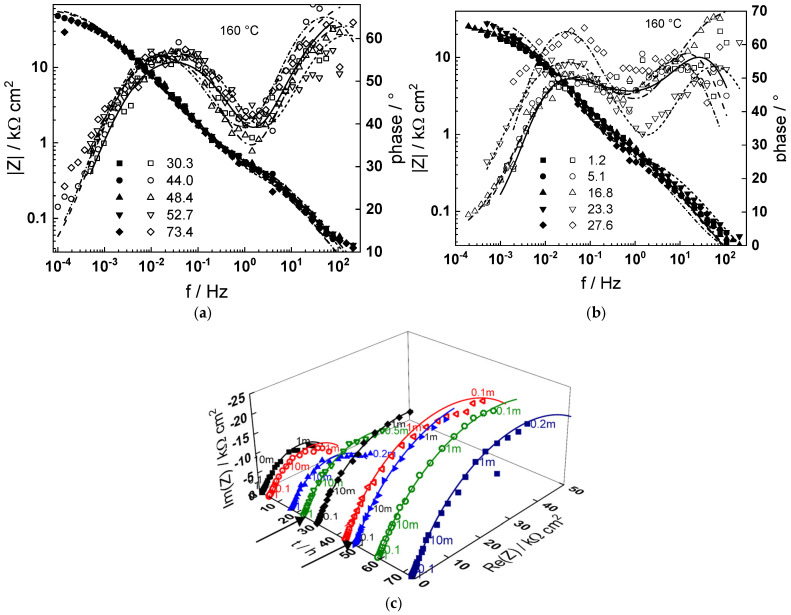
(**a**,**b**) Magnitude (left axis, full symbols) and phase angle (right axis, open symbols) of impedance as depending on frequency for different exposure times at 160 °C, (**c**) dependence of imaginary part of impedance on real part and time of exposure (parameter is frequency in Hz). Transitions to turbulent regime and back indicated with arrows. Points—experimental values, lines—best-fit calculation according to the MCM.

**Figure 5:**
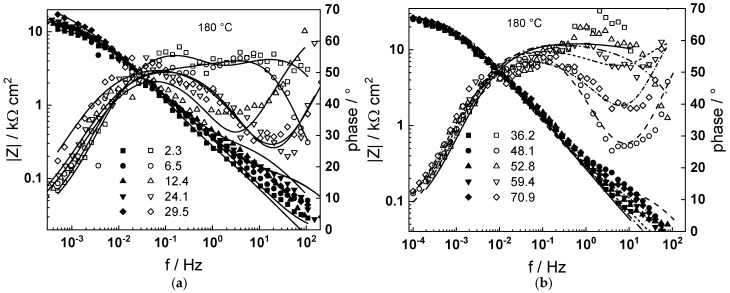

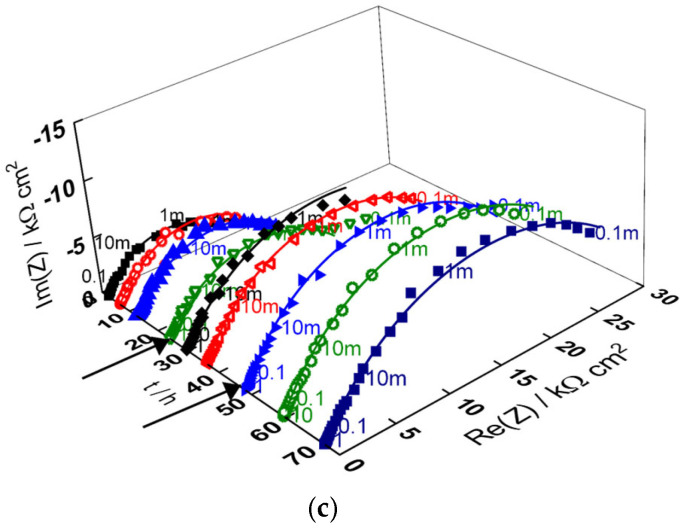
(**a**,**b**) Magnitude (left axis, full symbols) and phase angle (right axis, open symbols) of impedance as depending on frequency for different exposure times at 180 °C, (**c**) dependence of imaginary part of impedance on real part and time of exposure (parameter is frequency in Hz). Transitions to turbulent regime and back indicated with arrows. Points—experimental values, lines—best-fit calculation according to the MCM.

**Figure 6:**
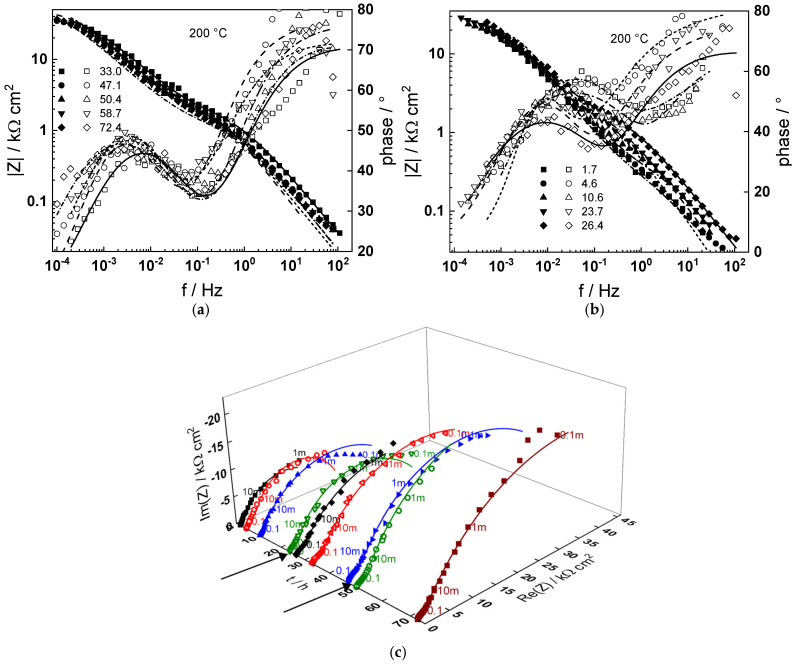
(**a**,**b**) Magnitude (left axis, full symbols) and phase angle (right axis, open symbols) of impedance as depending on frequency for different exposure times at 200 °C, (**c**) dependence of imaginary part of impedance on real part and time of exposure (parameter is frequency in Hz). Transitions to turbulent regime and back indicated with arrows. Points—experimental values, lines—best-fit calculation according to the MCM.

**Figure 7:**
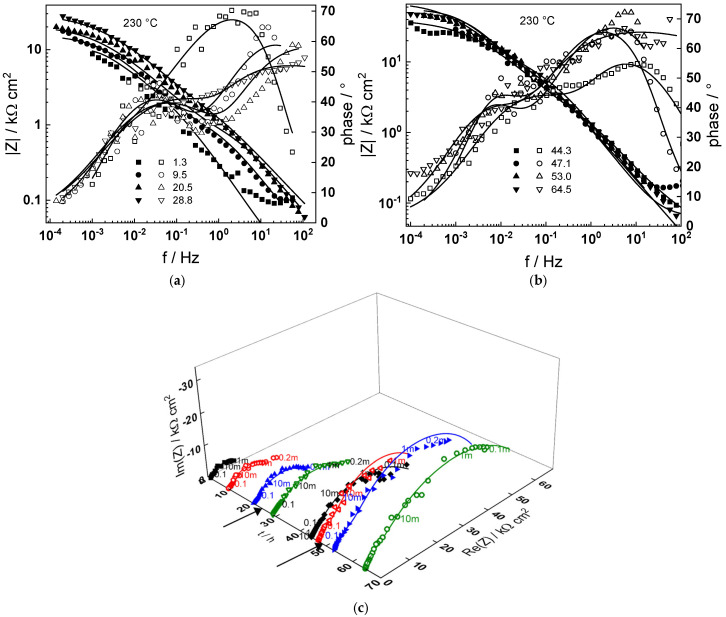
(**a**,**b**) Magnitude (left axis, full symbols) and phase angle (right axis, open symbols) of impedance depending on frequency for different exposure times at 230 °C, (**c**) dependence of imaginary part of impedance on real part and time of exposure (parameter is frequency in Hz). Transitions to turbulent regime and back indicated with arrows. Points—experimental values, lines—best-fit calculation according to the MCM.

**Figure 8:**
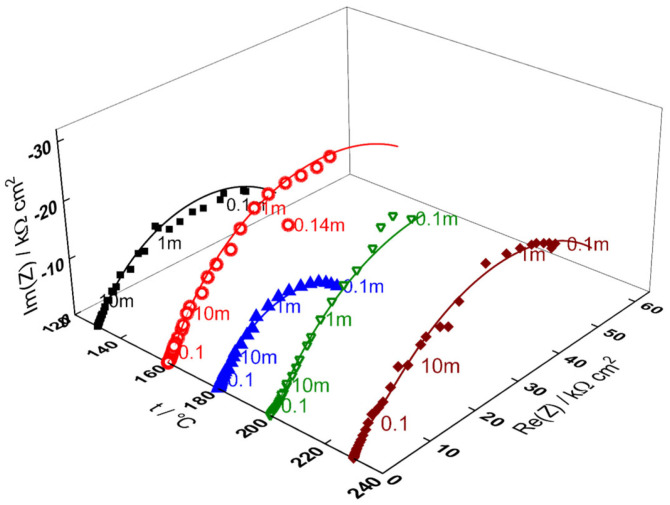
Dependence of imaginary part of impedance on real part and temperature at an exposure of 72 h (parameter is frequency in Hz). Points—experimental values, lines—best-fit calculation according to the MCM.

Impedance spectra as depending on temperature and exposure time are collected in Figure 3, Figure 4, Figure 5, Figure 6 and Figure 7 in both Bode (a, b) and complex plane coordinates (c). The influence of the transition to the turbulent regime is more evident at lower temperatures (130–180 °C). The impedance magnitude at frequencies approaching zero that approximates the polarization resistance of the corrosion process first slightly decreases and then increases during such a transition. After the return to transitional regime at 48 h, the increase in polarization resistance with exposure time is the more pronounced, the higher the temperature, and the polarization resistance at 72 h is the lowest at 180 and the largest at 230 °C (Figure 8).

Using the distribution of relaxation times method [23], four processes at different characteristic frequencies were detected in the spectra. The response of the electric properties of the oxide layer is located at ca. 10–100 Hz, interfacial charge transfer processes related to corrosion—at 0.5 to 5 Hz, and at still lower frequencies (1–10 mHz), two overlapped processes related to solid state transport of ionic defects in the oxide during its formation and corrosion release are observed.

To quantitatively interpret the impedance data, the transfer function derived on the basis of the MCM is employed in analogy to previous work [24]:

(1) $$\begin{array}{l} Z=R_{el}+Z_{ox}+Z_{F/S},\;Z_{F/S}=\frac{1}{j\omega C_{F/S}+R_{F/S}^{-1}},\;Z_{f}={\left(Z_{e}^{-1}+Z_{ion,O}^{-1}++Z_{ion,Fe}^{-1}\right)}^{-1},\\ Z_{e}\approx \frac{RT}{2j\omega FELC_{sc}}\ln\frac{\left[1+j\omega \varepsilon \varepsilon_{0}\left(\frac{RT}{F^{2}D_{e}}\frac{k_{2O}+k_{2Fe}}{k_{O}+k_{Fe}}\right)\exp\left(2\frac{F}{RT}EL\right)\right]}{1+j\omega \varepsilon \varepsilon_{0}\left(\frac{RT}{F^{2}D_{e}}\frac{k_{2O}+k_{2Fe}}{k_{O}+k_{Fe}}\right)},\\ Z_{ion,O}\approx \frac{RT}{4F^{2}k_{O}(1-\alpha )\left(1+\sqrt{1+\frac{4j\omega R^{2}T^{2}}{D_{O}F^{2}E^{2}}}\right)},\;\\ Z_{ion,Fe}\approx \frac{RT}{4F^{2}k_{Fe}(1-\alpha )\left(1+\sqrt{1+\frac{4j\omega R^{2}T^{2}}{D_{Fe}F^{2}E^{2}}}\right)} \end{array}$$

In these equations, *k_i_*, i = O, Fe, 2O, 2Fe are the rate constants of interfacial reactions of formation of magnetite-type oxide doped with Cr and Ni (M_3_O_4_) and iron oxidation to interstitial cations (${\mathrm{Fe}}_{i}^{••}$)

(2) $$\begin{array}{l} (\mathrm{Alloy}/\mathrm{Film})\;3m\overset{k_{O}}{\to }{3M}_{M}{(M}_{3}O_{4}{)\;+\;4V}_{O}^{••}+8e^{\prime }\\ (\mathrm{Alloy}/\mathrm{Film})\;\mathrm{Fe}\overset{k_{Fe}}{\to }M_{M}{+\mathrm{Fe}}_{i}^{••}+2e^{\prime }\\ {(\mathrm{Film}/\mathrm{Solution})\;4H}_{2}{O+4V}_{O}^{••}\overset{k_{2O}}{\to }{4O}_{O}{(M}_{3}O_{4}{)\;+\;8H}^{+}\\ {(\mathrm{Film}/\mathrm{Solution})\;\mathrm{Fe}}_{i}^{••}\overset{k_{2\mathrm{Fe}}}{\to }{\mathrm{Fe}}^{y+}+(y\text{-}2)e^{\prime } \end{array}$$

The charge transfer reactions are coupled by inward transport of oxygen via vacancies $V_{O}^{••}$ and outward transport of Fe via interstitial cations ${\mathrm{Fe}}_{i}^{••}$, as well as electrons (e′). The transport proceeds by solid state diffusion-migration characterized by the respective diffusion coefficients (*D_e_*, *D_O_*, and D_Fe_) and the field strength *E*. The rate constants of interfacial reactions are exponential functions of the potential drops at the respective interfaces, that (together with that in the oxide with thickness *L*) sum up to the corrosion potential.

(3) $$E=\phi_{A/F}+EL+\phi_{F/S},\;\phi_{F/S}=\alpha E+\phi_{F/S}^{0},\;\phi_{A/F}=(1-\alpha )E-EL.$$

The coupled reaction that consumes electrons from metal oxidation is the reduction in water. It is assumed to be a single step reaction with a charge transfer resistance R_F/S_ coupled to the capacitance of the double layer at the oxide/solution interface *C_F/S_*. The semiconductor properties of the oxide are associated with the space charge capacitance *C_sc_*, whereas *ε* and α are the dielectric constant of the oxide and the polarizability of the film/solution interface.

It is important to mention that since the study is conducted under controlled laboratory conditions, which may not fully replicate real-world operational environments, potential variations in water chemistry and mechanical stresses are not included in the model. The influence of stresses on interfacial reactions and transport processes in the oxide is currently under study and will be added to the model at a future stage.

**Figure 9:**
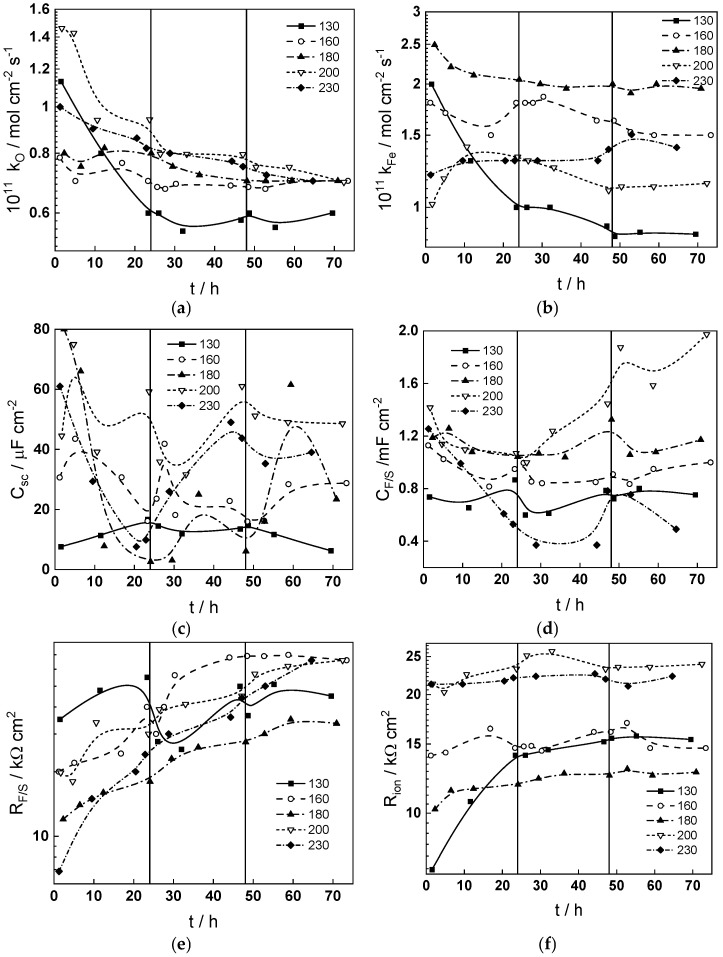

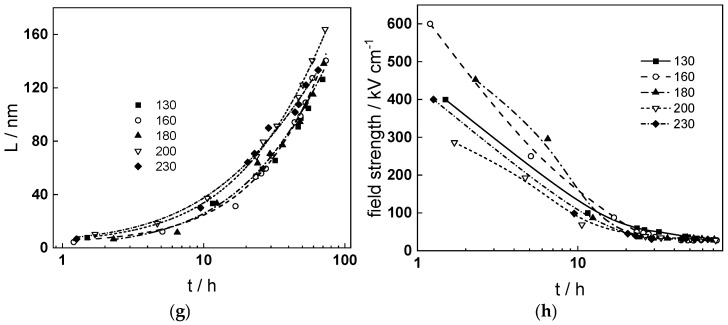
Dependences of the main kinetic and transport parameters estimated from regression of impedance data with respect to MCM equations on time and temperature: rate constants of oxide film formation (**a**) and Fe oxidation (**b**) at the alloy/film interface, space charge capacitance of the oxide (**c**) and capacitance of the film/solution interface (**d**), charge transfer resistance at the same interface (**e**), ion transport resistance (**f**), oxide film thickness (**g**) and field strength vs. film thickness and temperature (**h**). Vertical lines illustrate the transitions to turbulent regime and back.

The best-fit calculated values of the impedance as depending on temperature and time of exposure are shown in Figure 3, Figure 4, Figure 5, Figure 6, Figure 7 and Figure 8 with continuous lines and demonstrate the ability of the model to account successfully for both the magnitude and frequency distribution of the experimental data. The dependences of the parameters derived from the model on time and temperature are shown in Figure 9a–e. The ion transport resistance defined as the zero-frequency limit of the two ionic transport impedances is also presented

(4) $$R_{ion}=\underset{f\to 0}{\lim}\left(Z_{ion,O}+Z_{ion,Fe}\right)=\frac{RT}{8F^{2}(1-\alpha )\left(k_{O}+k_{Fe}\right)}.$$

The main observations regarding the effect of temperature, exposure time and flow rate on kinetic and transport parameter values are itemized as follows:

(1) The rate constants of the oxidation processes at the alloy/film interface (*k_O_* and *k_Fe_*, Figure 9a) in general decrease with time at constant temperature, the increase being more pronounced at the higher temperatures. Assuming that the system is in a quasi-steady state, oxide film formation and iron oxidation are balanced by film dissolution and cation ejection rates that are lower at higher temperatures due to the enhanced stability of the passive oxide. The effect of hydrodynamic conditions on these two parameters is comparatively small, but an increase of *k_Fe_* during the transition to turbulent regime is detected at 160 °C, indicating a transient increase in the rate of cation ejection (corrosion release) followed by a return to the steady state. The dependence of the rate constant *k_Fe_* at the end of exposure on temperature is non-monotonous and passes through a maximum at 180 °C, which correlates to the reported maxima in erosion–corrosion rates of low-alloy steel in nuclear plant steam generators [1,25,26]. This will be further elaborated upon in the Discussion section.
(2) Both capacitances—that of the space charge layer, *C_sc_*, and the interfacial double layer capacitance, *C_F/S_*, are influenced by the transition to turbulent regime at 24 h, and their values do not return to the original ones after the reverse transition at 48 h. This most probably means that the structure of the oxide/solution interface and the semiconducting properties of the surface film are altered by the increase in transient dissolution rate. The order of magnitude of *C_F/S_* (around 1–2 mF cm^−2^) point out to its identification as an adsorption pseudo-capacitance, most probably of an intermediate of the water reduction reaction. The values of both capacitances at 72 h increase slightly with temperature with maxima observed at 200 °C.
(3) The charge transfer resistance at the film/solution interface, *R_F/S_*, decreases at the transition to turbulent regime at 24 h, indicating a transient increase in the rate of the coupled reduction reaction provoked by an increase in corrosion rate. This decrease, is, however, of transient nature, and is overshadowed by the increase of *R_F/S_* with time at longer exposures. Once again, the temperature dependence of this resistance is non-monotonous with a minimum at 180 °C in agreement with the observed maximum of *k_Fe_* at the end of exposure, as discussed above. The effect of hydrodynamics on the ion transport resistance (*R_ion_*) is comparatively smaller, but similar to that on *R_F/S_*, which demonstrates the coupling of interfacial charge transfer and ion transport during corrosion. It is important to note that for most of the exposure time at a particular temperature, *R_ion_* < *R_F/S_*, i.e., charge transfer at the interface is slower than ion transport in the oxide. This is a somewhat unexpected result and an attempt to correlate this with the composition of the oxides is made in the Discussion section.
(4) Last but not least, the evolution of oxide thickness (*L*) with time follows a direct logarithmic law as derived in the MCM [24], and the field strength (***E***) in the oxide decreases with time reaching steady-state values after 20–30 h of exposure. The decrease in field strength can be explained by the formation of a space charge of interstitial iron cations and oxygen vacancies during the initial stages of film growth and corrosion release. Neither of these two parameters is influenced by the hydrodynamic conditions, and a minimum of thickness is observed at a temperature of 180 °C, indicating predominance of corrosion release over oxide formation around this temperature.

### 3.3. In-Depth Composition of the Oxides

**Figure 10:**
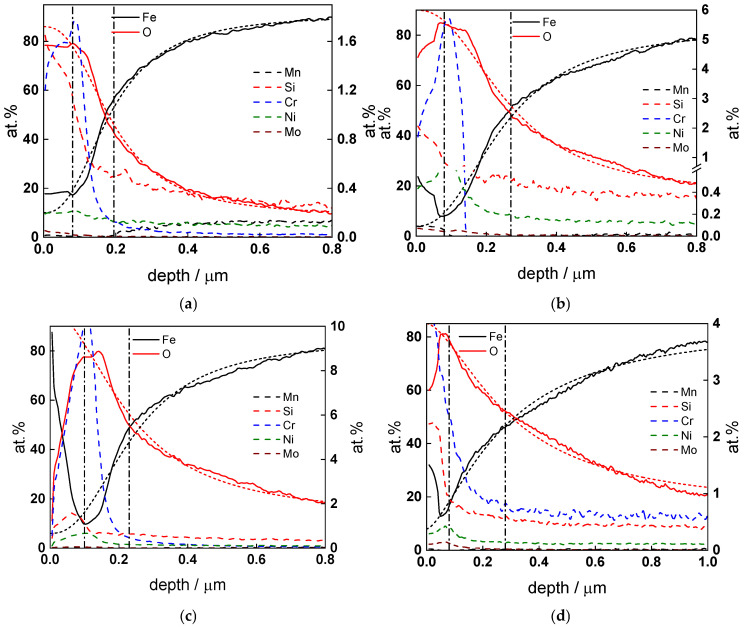

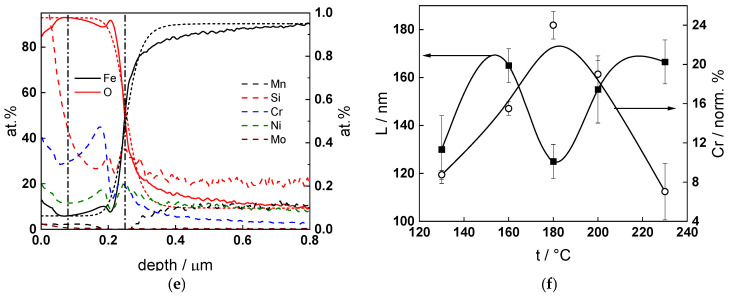
In-depth elemental composition (Fe, O, at. %, left axis, solid lines, Mn, Si, Cr, Ni and Mo, at.%, right axis. Dashed lines) of oxide layers formed on low-alloy steel at (**a**) 130, (**b**) 160, (**c**) 180, (**d**) 200 and (**e**) 230 °C for 72 h; thicknesses of contamination layer and oxide film (estimated by sigmoidal fits of O and Fe profiles, short dash lines) shown with vertical dash-dot lines; (**f**) temperature dependence of the mean thickness and the maximum Cr content normalized to all metal content (%).

GDOES depth profiles of the atomic concentrations of constituents for oxides formed for 72 h at all studied temperatures are collected in Figure 10a–e. The maximum Cr content in the oxide, normalized to the total metal content and averaged from three measurement points at each temperature, is compared to average film thickness as a function of temperature in Figure 10f.

It can be stated that the film thickness does not change significantly with temperature, the values being similar to those estimated from analysis of EIS data within the reproducibility limit (±10%), but a sufficiently pronounced minimum is observed at 180 °C. This minimum correlates well with a maximum in Cr content at the oxide/solution interface at that temperature, and enrichment of Ni and to a certain extent Mo is also detectable. These observations can be rationalized assuming that during the transition to turbulent regime at 24 h, enhanced dissolution of the outer layers of oxide and increase in the corrosion release rate (i.e., ejection of iron cations from the oxide) occurs, resulting in an enrichment of alloying elements, particularly Cr, that in turn leads to a decrease in dissolution rates and stabilization of the oxide. An attempt to rationalize all these experimental and calculation results in terms of the effect of flow rate, water chemistry and temperature is made in the Section 4.

## 4. Discussion

### 4.1. Influence of Temperature, Time and Hydrodynamic Conditions on Erosion–Corrosion Rate

The estimated kinetic parameters of oxide film formation and iron dissolution through the film (corrosion release) give the possibility to calculate the iron release rate, which in the present conditions is assimilated to the FAC rate [24]

(5) $$\begin{array}{l} \frac{dn_{Fe^{2+}}(t)}{dt}\approx k_{Fe}\exp\left[-\frac{2\alpha_{Fe}FE}{RT}L(t)\right]\\ L(t)=L_{0}+\frac{RT}{3\alpha_{O}FE}\ln\left[1+V_{m,ox}k_{O}\frac{3\alpha_{O}FE}{RT}e^{-\frac{3\alpha_{O}FE}{RT}L_{0}}t\right] \end{array}.$$

**Figure 11:**
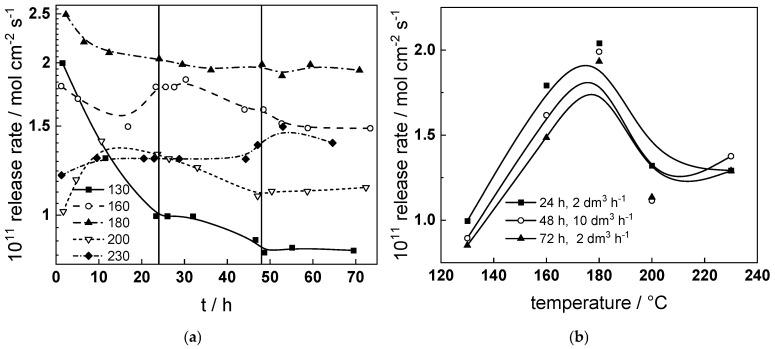
(**a**) dependence of iron release rate on time of exposure and temperature, transitions from volume flow rates of 2 to 10 and back to 2 dm^3^h^−1^ shown with solid lines; (**b**) release rate vs. temperature at three exposure times corresponding to the transitions to turbulent regime and back.

In the above equations, *L_O_* is the initial film thickness, *V_m,ox_*—its molar volume (44.5 cm^3^ mol^−1^ for Fe_3_O_4_), α_O_ and α_Fe_—the transfer coefficients of the oxide formation and iron oxidation reactions. Assuming that α_O_ = α_Fe_ = 0.1 is customary for interfacial reactions in high-temperature water electrolytes [27], the release rate is calculated and plotted vs. time and temperature in Figure 11a. The change in flow rate has at best only a transient influence on the release rate which decreases with time at constant temperature, reaching steady-state values for ca. 50–55 h. On the other hand, the temperature dependence of release rate is non-monotonous (Figure 11b), passing through a maximum at ca. 180 °C. This maximum is in accordance with several experimental studies and modeling approaches to FAC published in the recent literature [28,29]. As commented above, a minimum of both the charge transfer resistance of the coupled cathodic reaction and the rate constant *k_Fe_* of iron oxidation to generate interstitial cations are observed at the same temperature. In addition, a minimum of film thickness well correlated to a maximum in Cr content at the film/solution interface is also observed.

This indicates that the release rate is determined mainly by phenomena at the film/solution interface. At temperature of 180 °C, the flow rate increases to reach a turbulent regime most probably creates a transient effect on iron cation ejection rate, resulting in an increase in Cr content and predominance of corrosion release over oxide film formation in this temperature range.

### 4.2. Effect of Ammonia

**Figure 12:**
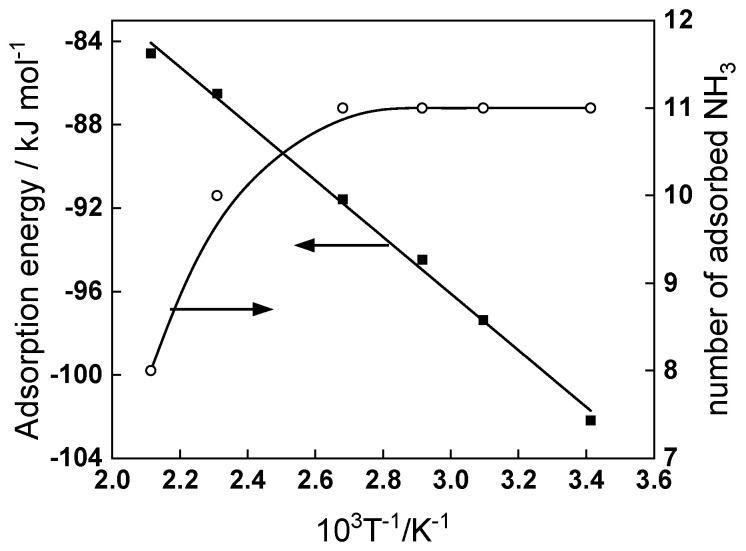
Adsorption energy (left axis) and number of adsorbed molecules (right axis) of NH_3_ on Fe_3_O_4_ in the temperature range 25–200 °C. Total number of ammonia molecules introduced in the simulation was 12 corresponding to a concentration of 0.18 mmol dm^−3^.

The adsorption of ammonia on Fe_3_O_4_ [111] in aqueous phase was studied in a companion paper [30] using molecular dynamics simulations in a similar temperature range (25–200 °C) at a pressure of 90 bars. Based on the results from these simulations, the adsorption energy of ammonia molecules at the surface of magnetite was calculated according to the equation:

(6) $$E_{ads}=E_{tot}-E_{slab/w}-E_{mol},$$

where *E_tot_* is the total energy of the system, *E_slab/w_*—the energy of the crystal slab in the aqueous medium and *E_mol_*—the energy of all the NH_3_ molecules before adsorption [31,32]. It is worth noting that at different temperatures the number of adsorbed molecules was found to be different despite the fact that the number of molecules introduced in the simulation was identical [30]. The adsorption energy and number of adsorbed molecules are presented in Figure 12 as a function of temperature.

The energy values are similar, albeit somewhat smaller, than the adsorption energies estimated by DFT for NH_3_ on tetragonal Fe-ion terminated Fe_3_O_4_ and Fe_2_O_3_ (114–137 kJ mol^−1^ [33,34,35]). The absolute values of the energy decrease with increasing temperature, which could be partly due to the smaller number of adsorbed molecules at the two highest temperatures [30] or the increase in the kinetic energy of the crystal with temperature [36]. Either way, the interaction between NH_3_ and the surface of Fe_3_O_4_ weakens at temperatures ≥ 160 °C. Recently, it has been reported that at low concentrations (<10^−4^ mol dm^−3^) ammonia acts as a blocking inhibitor with respect to carbon steel corrosion [37]. Thus, the weakened interaction of NH_3_ with the surface of magnetite can also contribute to the increase in corrosion release rate in the temperature range 160–180 °C. Further investigations are needed in order to experimentally estimate the adsorption energy of ammonia and other pH control agents in steam generators and its relation to the kinetic parameters of corrosion reactions on realistic corrosion product layers.

## 5. Conclusions

In the present paper, erosion–corrosion of low-alloy steel containing 0.6% Cr in high-ammonia, high-pH electrolyte at temperatures 130–230 °C that simulate startup and normal operation of a steam generator is studied by in situ EIS coupled to ex situ characterization of the formed corrosion layers using GDOES. Hydrodynamic conditions are varied between transition and turbulent regime by variations in the volume flow rate. EIS data are quantitatively interpreted using the MCM to obtain estimates of kinetic and transport parameters of the oxide formation and cation release processes.

The following main conclusions can be drawn from the experimental results and calculations:

(1) The influence of flow rate variation is clearly detected as a transient change in electrolyte conductivity and pH provoked by dissolution of the oxide and cation ejection. At temperatures lower than 200 °C, the polarization resistance first slightly decreases and then increases during flow rate transition to turbulent conditions. The polarization resistance at the end of exposure is found to be the lowest at 180 and the largest at 230 °C.
(2) The dependences of both the rate constant of iron oxidation and charge transfer resistance at the film/solution interface at the end of exposure on temperature pass through maxima at 180 °C, which correlates to the reported maxima in erosion–corrosion rates of low-alloy steel in nuclear plant steam generators.
(3) A sufficiently pronounced minimum in film thickness correlating with a maximum in Cr content at the oxide/solution interface is observed at 180 °C. This means that turbulent regime creates an increase of iron cation ejection rate, resulting in an increase in Cr content and predominance of corrosion release over film formation.
(4) The adsorption energy of ammonia on magnetite estimated from molecular dynamics simulations decreases with increasing temperature, i.e., the weakened interaction of NH_3_ with the surface of magnetite also contributes to the increase in corrosion release rate in the temperature range 160–180 °C.

## Data Availability

The original contributions presented in this study are included in the article. Further inquiries can be directed to the corresponding author.
